# “We Are Our Own Worst Enemies”: Workplace Bullying Among Nurses and Its Implications on Healthcare Workers and Job Performance: A Multi‐Facility Study in the Tamale Metropolis

**DOI:** 10.1002/nop2.70282

**Published:** 2025-07-20

**Authors:** Abubakari Wuni, Ajara Musah, Iddrisu Mohammed Sisala, Abdul Malik Abdulai, Letitia Chanayireh, Brenda Abena Nyarko, Hannah Buasilenu, Mudasir Mohammed Ibrahim, Sulemana Musah, Dorothy Azure, Nafisah Abdulai

**Affiliations:** ^1^ Department of Medicine for the Elderly (C6) Cambridge University Hospitals NHS Foundation Addenbrookes Hospital Cambridge UK; ^2^ Nurses' and Midwives' Training College Tamale Ghana; ^3^ Department of Midwifery and Women's Health School of Nursing and Midwifery, University for Development Studies Tamale Ghana; ^4^ Department of Health Science Regentropfen College of Health Science Bongo Ghana; ^5^ Department of Paediatrics and Child Health Tamale Teaching Hospital Tamale Ghana

**Keywords:** health workers, job performance, nurses, prevalence, workplace bullying

## Abstract

**Background:**

Workplace bullying is an important issue confronting the nursing profession, with victims described as being part of an oppressed group. The number of attacks and acts of violence that staff direct at each other in the workplace is alarmingly high and cannot be ignored.

**Aim:**

This study assessed the prevalence and impact of workplace bullying of nurses by other nurses among those working in three major hospitals in the Tamale Metropolis, Ghana.

**Methods:**

Data for this study were collected from Tamale Teaching Hospital, Tamale Central Hospital, and Tamale West Hospital using a descriptive cross‐sectional multi‐facility study design with a quantitative approach to data collection. A proportionate stratified random sampling technique was used to recruit 338 nurses from the three hospitals. A structured questionnaire was used to collect data, following approval from the Tamale Teaching Hospital Research and Development Unit and the Northern Regional Health Directorate, from January 2022 to March 2022. Stata for Windows V16.0 was used to analyse the data. Bivariate and multivariable logistic regression models were used to explore the factors associated with the prevalence of perceived workplace bullying among nurses. Confidence intervals were computed at a 95% confidence level, and a *p*‐value of 0.05 or less was considered statistically significant.

**Results:**

The majority of respondents (85.5%) indicated they had observed workplace bullying before, and 50.6% had witnessed the bullying of a nurse by another nurse. A little over one‐third had been victims of workplace bullying. Females constituted a higher proportion of both the perpetrators (53.0%) and the main targets (80.2%) of workplace bullying. Additionally, 34.6% reported having the intention to travel abroad to practise nursing as a result of observing bullying or being victims themselves. Multivariable analysis showed that the odds of experiencing workplace bullying were 63% lower among nurses working in the surgical ward compared to those in the Outpatient Department (AOR: 0.37, 95% CI: 0.15–0.91, *p* = 0.030).

**Conclusion:**

This study revealed that workplace bullying is prevalent among nurses in the three main hospitals within the Tamale Metropolis. Most respondents had witnessed workplace bullying, and a little over one‐third had been victims themselves. Workplace bullying is a measurable issue that negatively affects nurses' mental health and job performance. Therefore, nursing leaders should organise regular sensitisation programmes to raise awareness of the impact of workplace bullying. Additionally, hospital management should encourage nurses to report instances of bullying, establish disciplinary committees to address such cases, punish offenders, and protect those who witness or are victims of bullying.

## 1 Introduction

Workplace bullying is a common psychosocial risk factor that is prevalent in almost all workplaces around the world (Teo et al. 2021). It is a major public issue that is receiving increasing attention, having been recorded in various countries and across a wide range of professions (Al‐Ghabeesh and Qattom 2019). Workplace bullying encompasses behaviours such as harassment, causing offence, and social exclusion, making it a highly severe manifestation of violence (Zhang et al. 2023).

Over the years, a substantial and growing body of evidence has accumulated, demonstrating that workplace bullying is a widespread and costly occurrence (Bambi et al. 2018). The frequency of workplace bullying can vary significantly, ranging from 1.3% to as high as 96%. However, there is substantial evidence indicating that workplace bullying is a common issue in the nursing profession and across various nations (Hosier et al. 2023). Nurses in healthcare settings are particularly susceptible to workplace bullying, with reported prevalence rates as high as 20%–30%. This issue is especially prevalent in healthcare sectors with a hierarchical organisational culture (Yun and Kang 2018).

Many definitions have been proposed that highlight the characteristics of workplace bullying among nurses. Workplace bullying is “a harmful phenomenon, characterized by repeated actions and practices directed against one or more workers, which may be carried out deliberately or unconsciously. It causes a sense of humiliation, offense, and anguish that interferes with the victims' work performance and/or leads to an unpleasant work environment” (João and Portelada 2023). Workplace bullying can be overt or covert, and may be physical (involving direct bodily contact with the target, such as hitting and slapping), verbal (involving the use of words such as gossiping and shouting), or cyber (through email, text messages, and social media) (Nwaneri and Onoka 2017; Jang et al. 2023; Plos et al. 2022). Other common forms of workplace bullying include non‐verbal innuendo, undermining activities, withholding information, sabotage, gloating, backstabbing, failure to respect privacy, and broken confidences (Al‐Ghabeesh and Qattom 2019).

Bullying is recognised as a widespread public health issue that transcends borders, affecting various workplaces worldwide and posing significant risks to individuals' health and overall welfare. This behaviour can disturb interpersonal dynamics within the workplace, resulting in detrimental effects on productivity and creating an unhealthy work atmosphere (Abdulkarim and Subke 2023). In healthcare settings, particularly among nursing staff, these harmful behaviours take on unique forms and consequences that warrant focused attention (Smith et al. 2020).

Nurse administrators are aware of the prevalence of workplace bullying, and research has been carried out to investigate related concerns. Those particularly susceptible to bullying include women, new employees, individuals with disabilities, and those from marginalised communities (Aunger et al. 2023). The abuse of power and mobilisation of bias are central components of workplace bullying (Mannix‐McNamara 2021).

The dynamics driving bullying behaviours within nursing settings are influenced by a combination of enabling, motivating, and precipitating factors that interact to perpetuate bullying conduct. Enabling factors, such as real or perceived power imbalances, create environments conducive to bullying behaviours (Tuckey et al. 2024). Motivations for bullying behaviours in nursing can stem from a variety of sources, including power differentials, organisational culture, and individual characteristics. The hierarchical abuse of power in work organisations has been identified as a significant factor contributing to bullying behaviours (Quinn et al. 2024). Additionally, the rewards associated with bullying, such as financial gain and power, can perpetuate such behaviours over time (Hutchinson et al. 2010; Xu et al. 2019). Precipitating factors trigger the onset of bullying behaviours, leading to the persistence and escalation of mistreatment over time, and may include economic conditions, workload, lack of interpersonal skills, lack of management skills, and nurses not feeling empowered (Ko et al. 2020).

Workplace bullying is closely linked with a desire to leave the profession and is associated with increased absenteeism and a tendency to leave healthcare facilities (Serafin and Czarkowska‐Pączek 2019; Homayuni et al. 2021). Nurses who have experienced bullying often report low job satisfaction and a decline in work performance, which can result in more medical errors and poor patient outcomes (Homayuni et al. 2021; Jang et al. 2023). Additionally, exposure to bullying has been linked to mental health issues among nurses globally, as well as physical and psychological problems reported by many victims (Colaprico et al. 2023; Abdulkarim and Subke 2023). Many bullying victims have been recognised to demonstrate symptoms of Post‐Traumatic Stress Disorder (PTSD), and some have reportedly attempted suicide (Lu et al. 2023).

There have been relatively few studies that consider the prevalence of workplace bullying in the nursing profession. Overseas, workplace bullying is recognised as one of the violent acts experienced by nurses, and laws or regulations have been issued to address it (Choi and Noh 2020). The obvious negative impacts of bullying on health professionals necessitate early intervention and staff recognition of what is occurring in order to prevent continued bullying (Lever et al. 2019; Ullah et al. 2023). Given that the prevalence of bullying is higher in healthcare institutions, this study assessed the prevalence and impact of workplace bullying among nurses working in three major hospitals in the Tamale Metropolis, Ghana.

## 2 Materials and Methods

### 2.1 Study Design

A descriptive cross‐sectional design with quantitative data collection was employed to gather data from nurses working in the three main hospitals within the Tamale Metropolis.

### 2.2 Study Area and Population

The study was conducted in three hospitals located within the Tamale Metropolis. Tamale is the capital city of the Northern Region of Ghana. Though other healthcare facilities provide nursing and medical care within the metropolis, these three are the largest. Hospital A provides tertiary care and serves as a referral centre for the five Regions of the North and other surrounding regions. Hospital B is a hospital within the Tamale Metropolis that provides healthcare for the metropolis and beyond. Hospital C is currently a referral hospital for metropolitan sub‐district health centres providing 24‐h services. The three hospitals have a total registered nursing population of 2166, which constitutes the target population. Data were collected from the Outpatients Department (OPD), Medical and Surgical wards, Paediatrics ward, and Operating Theatres.

### 2.3 Sample Size and Sampling Procedure

**FIGURE 1:**
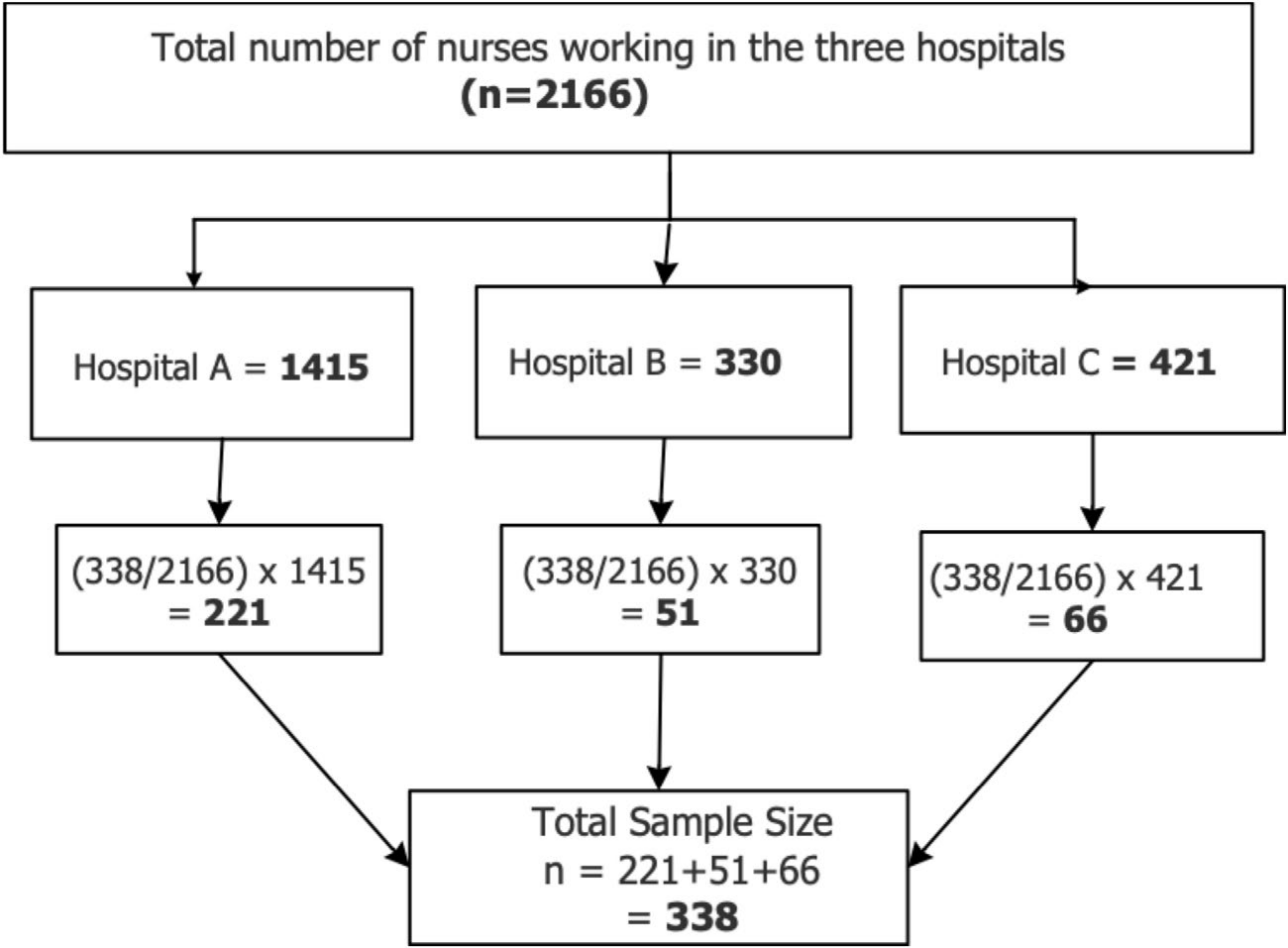
Flow chart for sample size calculation.

The three hospitals had a total registered nursing population of 2166. A sample size of 338 was determined for the study using Yamane's (1967) formula for determining sample size based on a known population: $\frac{N}{1+N{\left(e\right)}^{2}}$. The study employed a proportionate stratified random sampling technique [Sample Size/Population Size × Stratum Size] to select nurses from the three hospitals to be part of the study. The total sample size was allocated proportionally to each hospital based on the size of its nursing population (See Figure 1). A simple random sampling method was then used to select respondents within each hospital. Nurses in each hospital were assigned a unique identifier, and a random number generator in SAS JMP was utilised to select the required number of respondents. This approach ensured that the selected sample was representative of the entire population of nurses across the three hospitals.

### 2.4 Inclusion and Exclusion Criteria

The study included registered nurses who had worked in the hospitals for at least six months and consented to be part of the study. Student nurses who were on their clinical practicum and qualified nurses doing their mandatory national service were excluded.

### 2.5 Data Collection Instrument

A structured questionnaire was used to collect data from the nurses working in the hospitals. This questionnaire was adopted from Einarsen et al. (2009) and Nwaneri and Onoka (2017), and modified to reflect the objectives of the study. The instrument included a 16‐item modified Negative Acts Questionnaire (NAQ) and a 15‐item General Health Questionnaire (GHQ), in addition to other relevant questions. A Likert scale response format was used to assess the frequency of exposure. The questionnaire had five sections: Section A—Demographic Characteristics of Nurses in the three hospitals (9 items); Section B—Nurses' perceived exposure (prevalence) of workplace bullying (6 items); Section C—Negative Acts Questions (NAQ) (16 items); Section D—Perceived characteristics of the main perpetrators and targets of workplace bullying (2 items); and Section E—General Health Questions (GHQ) to assess the impact of workplace bullying among nurses (15 items). The questionnaires were serially numbered to facilitate easier data entry and analysis.

### 2.6 Validity and Reliability

The questionnaire underwent peer review by the authors and other experts to evaluate its appropriateness and relevance. They critiqued its content to ensure content validity. It was then pretested among 35 nurses from two smaller facilities in the city to assess its consistency and accuracy. No ambiguities were found in the questions during pretesting, so the original format was retained. Validity and reliability of the Likert scale items were evaluated through Confirmatory Factor Analysis (CFA) using composite reliability and convergent validity measures. Composite reliability values ranged from 0.94 to 0.95, indicating high internal consistency across constructs. Convergent validity was confirmed by the Average Variance Extracted (AVE), which ranged from 0.52 to 0.58, exceeding the recommended threshold of 0.50 and indicating satisfactory validity.

### 2.7 Data Collection Procedure

Data collection began after authorisation was obtained from the Research and Development units of the three hospitals. The process took place from January to March 2022 using the structured questionnaire. The questionnaires were administered to nurses in their respective wards after obtaining both verbal and written consent. Completed questionnaires were reviewed for accuracy and consistency, then stored in sealed envelopes. Each questionnaire was pre‐coded with an identification number for easy tracking. Research assistants provided neutral and clear explanations to respondents as needed. Confidentiality was maintained throughout the process. All respondents were taken through the informed consent process, and clarifications were provided to those requiring further explanation.

### 2.8 Data Analysis

Descriptive statistics were performed for categorical data and presented in tables. The prevalence of perceived workplace bullying among nurses was assessed using the Negative Acts Questionnaire‐Revised (NAQ‐R), where 16 questions were scored. Respondents were scored 0 if they never experienced a negative act per week for at least six months, and 1 if they experienced it occasionally, monthly, weekly, or daily. Those subjected to at least one negative act were classified as bullied, while those who did not experience any were classified as not bullied (bullied = 1; not bullied = 0). Bivariate and multivariable logistic regression models were used to explore factors associated with perceived workplace bullying. Confidence intervals were calculated at a 95% level, and a *p*‐value < 0.05 was considered statistically significant. Statistical analyses were conducted using Stata Windows V16.0.

### 2.9 Ethical Consideration

Ethical approval was granted to carry out this study by the hospitals, with the protocol numbers REDACTED approved by their respective ethics committees [REDACTED]. The two primary hospitals and the tertiary hospital officially approved the conduct of the research on January 20, 2022, and January 25, 2022, respectively. Before administering the questionnaire, authorisation was obtained from the ward managers, as well as informed consent from the nurses. The nurses who agreed to participate in the study were assured of privacy and confidentiality throughout the process.

## 3 Results

### 3.1 Socio‐Demographic Characteristics of Respondents

Table 1 presents the socio‐demographic characteristics of respondents. Most were aged 30–39 years (49.7%), females formed a slightly higher proportion (51.2%), and more than half were married (59.2%). The majority had worked for 1–5 years (63.6%). A higher proportion of respondents were registered general nurses (86.1%), 66.6% were junior staff, and about one‐third (36.7%) worked in the surgical ward. The majority of respondents worked at Hospital A (65.7%).

**TABLE 1:** Socio‐demographic characteristics.

Variable	Frequency (*n* = 338)	Percent (%)
Age (years)		
20–29	161	47.6
30–39	168	49.7
40–49	8	2.4
50–59	1	0.3
Sex		
Female	173	51.2
Male	165	48.8
Marital status		
Single	135	39.9
Married	200	59.2
Divorced	3	0.9
Work experience		
1–5	215	63.6
6–10	87	25.7
11–15	36	10.7
Cadre		
Registered general nurse	291	86.1
Enrolled Nurse	42	12.4
Community Health Nurse	5	1.5
Rank		
Junior cadre	225	66.6
Middle cadre	98	29.0
Senior cadre	15	4.4
Department		
Surgical ward	124	36.7
Paediatrics	84	24.8
Medical ward	77	22.8
OPD	42	12.4
Other^a^	11	3.3
Hospital		
Hospital A	222	65.7
Tamale C	66	19.5
Hospital B	50	14.8
Religious affiliation		
Islam	176	52.1
Christian	156	46.1
Traditional	6	1.8

*Note:* Field Survey (2022).

Abbreviation: OPD, Outpatient Department.

^a^ Labour ward, Theatre, Emergency ward.

### 3.2 Prevalence of Perceived Workplace Bullying Among Nurses

The majority of respondents indicated that they had observed workplace bullying before (85.5%). Most (66.9%) indicated that bullying of nurses by nurses existed in their hospital, while 52.4% indicated it existed among nurses in their ward. Furthermore, slightly more than half (50.6%) had witnessed the bullying of a nurse by another nurse. Regarding personal experience, 36.7% of respondents indicated that they had been victims of workplace bullying, while 63.3% stated that they had not. Among those who had experienced bullying, 57.3% reported the incident to the authorities (Table 2).

**TABLE 2:** Prevalence of perceived workplace bullying among nurses.

Variable	Frequency (*n* = 338)	Percent (%)
Have heard of workplace bullying before		
No	49	14.5
Yes	289	85.5
Workplace bullying of nurses by nurses exist in my hospital		
No	112	33.1
Yes	226	66.9
Workplace bullying of nurses by nurses exists in my ward		
No	161	47.6
Yes	177	52.4
Ever witnessed workplace bullying of a nurse by another nurse		
No	167	49.4
Yes	171	50.6
Respondent has been a victim of workplace bullying		
No	214	63.3
Yes	124	36.7
Victims of workplace bullying of nurses by nurses reported incidence to the authorities (*n* = 124)		
No	53	42.7
Yes	71	57.3

*Note:* Field Survey (2022).

### 3.3 Frequency of Workplace Bullying Among Nurses Within the Past Six Month

Of the 338 respondents, 84.6% indicated that they had observed at least one of the negative behaviours assessed for bullying either weekly or daily during the past six months. Negative behaviours that occurred most frequently in the past six months were having opinions ignored (47.6%), followed by withholding information (46.2%), spreading gossip and rumours (45.0%), and the least observed was sexual assault or rape (15.1%). Spreading gossip and rumours was the most observed daily behaviour (16.9%) (Table 3).

**TABLE 3:** Frequency of workplace bullying among nurses within the past six months.

Variable	Weekly *n* = %	Daily *n* = %	Total *n* = %
Ignored or having opinions ignored	108 (31.9)	53 (15.7)	161 (47.6)
Withholding information which affects performance	104 (30.8)	52 (15.4)	156 (46.2)
Spreading of gossip and rumours about you	95 (28.1)	57 (16.9)	152 (45.0)
Have been demeaned before	103 (30.5)	43 (12.7)	146 (43.2)
Been ordered to work below the level of your competence	96 (28.4)	37 (10.9)	133 (39.3)
Having been insulted and bad words thrown at	93 (27.5)	36 (10.7)	129 (38.2)
Been exposed to an unmanageable workload	89 (26.3)	34 (10.1)	123 (36.4)
Repeated reminders of your errors and mistakes	80 (23.7)	33 (9.7)	113 (33.4)
Persistent allegations and false accusations	71 (21.0)	33 (9.8)	104 (30.8)
Been humiliated or ridiculed in the workplace	71 (21.3)	32 (9.5)	103 (30.8)
Have been threatened by staff	70 (20.7)	26 (7.7)	96 (28.4)
Given task of unrealistic deadlines	70 (20.7)	22 (6.5)	92 (27.2)
Been subject of excessive teasing and sarcasm	63 (18.6)	22 (6.5)	85 (25.1)
Threats of violence or actual abuse	56 (16.6)	24 (7.1)	80 (23.7)
Physically attacked	41 (12.1)	25 (7.4)	66 (19.5)
Sexual assault or rape	29 (8.6)	22 (6.5)	51 (15.1)

*Note:* Field Survey (2022).

### 3.4 Characteristics of the Main Perpetrators and Targets of Workplace Bullying

Table 4 shows that females formed a higher proportion of the perpetrators (53.0%) and main targets (80.2%) of workplace bullying. Most of the main perpetrators were senior staff (55.3%), whereas the majority of the main targets were junior staff (90.8%).

**TABLE 4:** Respondent's view of the characteristics of the main perpetrators and targets of workplace bullying among nurses.

Variable	Main perpetrators *n* (%)	Main targets *n* (%)
Gender		
Female	179 (53.0)	271 (80.2)
Male	159 (47.0)	67 (19.8)
Cadre		
Junior cadre	8 (2.4)	307 (90.8)
Middle cadre	143 (42.3)	30 (8.9)
Senior cadre	187 (55.3)	1 (0.3)

*Note:* Field Survey (2022).

### 3.5 Impact of Workplace Bullying Among Nurses on the General Health, Job Performance and Retention

Of the 338 respondents, 34.6% strongly agreed that they had the intention to travel abroad to practise nursing as a result of observed bullying or being victims themselves. Almost a quarter (24.6%) felt powerless to change their bullying situation, 21.6% strongly agreed that the bullying had left a poor working relationship between themselves and their colleagues, and 19.5% were left with an inferiority complex (Table 5).

**TABLE 5:** Impact of workplace bullying among nurses on the general health, job performance, and retention.

Variable	Strongly Disagree *n* (%)	Disagree *n* (%)	Undecided *n* (%)	Agree *n* (%)	Strongly agree *n* (%)
Anger	39 (11.5)	57 (16.9)	47 (13.9)	144 (42.6)	51 (15.1)
Anxiety	37 (10.9)	56 (16.6)	80 (23.7)	129 (38.2)	36 (10.6)
Emotional outburst or shutdown	26 (7.7)	51 (15.1)	73 (21.6)	137 (40.5)	51 (15.1)
Frustration	28 (8.3)	53 (15.7)	59 (17.5)	147 (43.5)	51 (15.1)
Headache/Migraine	26 (7.7)	78 (23.1)	91 (26.9)	110 (32.5)	33 (9.8)
Dread going to work	36 (10.6)	92 (27.2)	89 (26.3)	96 (28.4)	25 (7.4)
Took some days off due to emotional trauma associated with bullying	59 (17.5)	106 (31.4)	73 (21.6)	63 (18.6)	37 (10.9)
Intention to leave the nursing profession	57 (16.9)	82 (24.2)	66 (19.5)	75 (22.2)	58 (17.2)
Job dissatisfaction	36 (10.6)	61 (18.1)	59 (17.5)	119 (35.2)	63 (18.6)
Low self‐esteem	38 (11.2)	82 (24.3)	64 (18.9)	91 (26.9)	63 (18.6)
Medication errors and other mistakes while working	49 (14.5)	74 (21.9)	56 (16.6)	102 (30.2)	57 (16.8)
Poor working relationship as team between colleagues and I	39 (11.5)	75 (22.2)	47 (13.9)	104 (30.8)	73 (21.6)
Inferiority complex	39 (11.6)	81 (24.0)	64 (18.9)	88 (26.0)	66 (19.5)
Feel powerless to change my situation	50 (14.8)	68 (20.1)	60 (17.7)	77 (22.8)	83 (24.6)
Intention to travel abroad to practice nursing	45 (13.3)	59 (17.5)	61 (18.0)	56 (16.6)	117 (34.6)

### 3.6 Factors Associated With Prevalence of Perceived Workplace Bullying Among Nurses

We conducted bivariate and multivariate binary logistic regression analyses to examine the association between department, hospital, and the perceived prevalence of workplace bullying. At the bivariate level, the department and hospital of work were significantly associated with perceived workplace bullying. In the multivariate analysis controlling for potential confounding variables including age, sex, marital status, work experience, cadre, rank, and religious affiliation, only the association between department and perceived prevalence of workplace bullying remained significant. The multivariate analysis further revealed that the odds of perceived workplace bullying were 63% lower among nurses working in the surgical ward (AOR = 0.37, 95% CI: 0.15–0.91, *p* = 0.030) compared to those in the outpatient department (Table 6).

**TABLE 6:** Factors associated with prevalence of perceived workplace bullying among nurses.

Variable	COR	95% CI	*p*	AOR^a^	95% CI	*p*
Age (years)						
20–29	1			1		
30–39	0.89	0.56–1.39	0.600	0.64	0.33–1.24	0.187
40–49	1.32	0.29–5.87	0.717	0.87	0.14–5.44	0.883
50–59	1.52	0.06–37.96	0.798	1.79	0.07–47.64	0.728
Sex						
Male	1			1		
Female	1.29	0.82–2.02	0.266	1.21	0.75–1.98	0.432
Marital status						
Single	1			1		
Married	1.11	0.70–1.75	0.662	1.06	0.60–1.87	0.850
Divorced	4.00	0.20–79.15	0.362	6.47	0.30–67.68	0.234
Work experience						
1–5	1			1		
6–10	1.41	0.83–2.39	0.209	1.46	0.72–2.97	0.292
11–15	1.53	0.71–3.29	0.277	1.18	0.40–3.53	0.762
Cadre						
Registered general nurse	1			1		
Enrolled Nurse	1.38	0.68–2.78	0.367	1.40	0.64–3.05	0.401
Community Health Nurse	6.22	0.34–113.64	0.217	4.59	0.23–90.55	0.316
Rank						
Junior cadre	1			1		
Middle cadre	1.46	0.88–2.43	0.143	1.55	0.83–2.92	0.170
Senior cadre	3.35	0.84–13.25	0.085	2.07	0.42–10.15	0.369
Department						
OPD	1			1		
Surgical ward	0.34	0.14–0.82	0.016^a^	0.37	0.15–0.91	0.030^a^
Paediatrics	0.36	0.14–0.88	0.026^a^	0.41	0.16–1.05	0.065
Medical ward	0.39	0.15–0.97	0.042^a^	0.42	0.16–1.08	0.073
Others	0.25	0.06–0.99	0.049^a^	0.20	0.04–1.02	0.053
Hospital						
Hospital A	1			1		
Hospital C	1.88	1.00–3.52	0.050^a^	1.45	0.74–2.86	0.276
Hospital B	0.71	0.39–1.32	0.287	0.53	0.26–1.06	0.074
Religious affiliation						
Christian	1			1		
Islam	0.73	0.46–1.15	0.172	0.72	0.44–1.16	0.178
Traditional	5.99	0.22–65.36	0.226	10.35	0.46–87.45	0.141

Abbreviations: AOR, Adjusted odds ratio; COR, Crude odds ratio.

^a^ All variables were adjusted in the multivariate model.

## 4 Discussion

The aim of the study was to assess the prevalence and impact of workplace bullying among nurses working in three major hospitals in the Tamale Metropolis, Ghana. Workplace bullying has been found to be prevalent in virtually all nursing workplaces. About 36.7% of the nurses reported being victims of bullying by fellow nurses among the 338 respondents. This is inconsistent with the results of Attia et al. (2020), who affirmed that the majority of their study population experienced bullying at the workplace. The discrepancy in findings could be attributed to differences in study settings. The current study was a multifacility investigation, whereas the study by Attia et al. (2020) was limited to a single facility.

The study revealed that a greater proportion of respondents had observed workplace bullying, acknowledged its existence among nurses in their hospitals, and indicated it was present among nurses in their wards. This result aligns with a study by Nwaneri and Onoka (2017), who reported that a majority of respondents acknowledged the existence of workplace bullying by nurses in their hospitals and wards and had also witnessed and experienced bullying in their respective units. This is also consistent with the findings of Attia et al. (2020), who affirmed that the majority of the study population experienced workplace bullying. These similarities could be attributed to the fact that bullying in hospitals is not peculiar to a particular country but is a global issue. Additionally, some nurses may be unaware of the behaviours that constitute workplace bullying or how it manifests; hence, they may be subjected to it without realising it.

The current study showed that 84.6% of respondents indicated they had observed at least one of the negative behaviours associated with bullying either weekly or daily during the past six months. The most frequently occurring negative behaviours in the past six months included having opinions ignored (47.6%), withholding information (46.2%), and spreading gossip and rumours (45.0%), with the least observed being sexual assault or rape (15.1%). Spreading gossip and rumours was the most commonly observed daily behaviour (16.9%). This resonates with a study by Etienne (2014), who found that 4% of respondents were victims of daily bullying, 12% were bullied weekly, and 16% received threats of violence or physical attacks monthly. It is also worth noting that Etienne (2014) reported that 35% of respondents were being ignored. This is consistent with findings from Nwaneri and Onoka (2017), Yahaya et al. (2012), Homayuni et al. (2021), and Edmonson and Zelonka (2019), who reported daily observations of behaviours such as backbiting, gossiping, invasion of privacy, excessive workload without supervision, humiliation in front of others, and unfair allocation of duties and postings.

This study reveals that workplace bullying among nurses is primarily perpetrated by and targeted at female nurses. This may be explained by relational hostility, which causes females to more frequently bully other females. Thus, female bullies in the workplace are more likely to target other females. Most of the main perpetrators were senior staff (55.3%), while the majority of the main targets were junior staff (90.8%). Consistent with previous studies (Serafin and Czarkowska‐Pączek 2019; Bambi et al. 2018), female nurses were found to be more frequently bullied than their male counterparts. These studies also found that senior and experienced nurses were more likely to bully junior and less experienced colleagues. In line with the current study, Homayuni et al. (2021) also reported that young nurses and those with less professional experience were more likely to be bullied. Serafin and Czarkowska‐Pączek (2019) demonstrated that nurses new to the workforce are more susceptible to bullying because they are often younger, less experienced, unfamiliar with their new roles, and less knowledgeable about unit cultural norms than their seasoned counterparts. These similarities may be due to the fact that increasing age in a profession is associated with greater experience and a lower likelihood of being bullied. However, this contrasts with the findings of Homayuni et al. (2021), who reported that males were more exposed to bullying behaviours. This could be attributed to the predominance of females in the current study population.

Workplace bullying significantly impacts nurses' overall health, job performance, and retention. Most nurses agreed that the effects of workplace bullying include anger, anxiety, emotional outbursts, frustration, job dissatisfaction, and feelings of inferiority. The results of this study demonstrate that workplace bullying negatively affects nurses' social and psychological well‐being. Additionally, most nurses strongly agreed that they had the intention to migrate abroad to practise nursing. This indicates that workplace bullying is a major factor driving nurse migration overseas (Nwaneri and Onoka 2017). It is possible that respondents believe that supposedly better working conditions in hospitals in developed countries could reduce the prevalence of workplace bullying. These findings are in line with those of Chowdhury et al. (2023) and Sauer and McCoy (2018), who reported that nurses who experienced bullying were more likely to migrate and that bullying leads to job dissatisfaction, depression, and anxiety. This study also supports previous findings that bullying leads to poor staff motivation and concentration (Mensah and Mpaun 2024), decreased productivity (Al‐Ghabeesh and Qattom 2019), and reduced commitment to work, as well as strained relationships with patients, managers, and colleagues (Perez 2019). Our study demonstrates that workplace bullying is one of the reasons Ghanaian nurses choose to practise nursing in other countries, particularly the United States, the United Kingdom, and Canada.

Additionally, the majority of respondents (85.5%) indicated that they had observed workplace bullying before, and 50.6% had witnessed the bullying of a nurse by another nurse. A little over one‐third had been victims of workplace bullying. Females formed a higher proportion of both perpetrators (53.0%) and targets (80.2%) of bullying. Furthermore, 34.6% reported an intention to travel abroad to practise nursing as a result of either observing or experiencing bullying. A multivariable analysis showed that the odds of perceived workplace bullying were 63% lower among nurses working in surgical wards compared to those in outpatient departments, indicating a lower exposure to bullying behaviours in surgical settings. This finding aligns with existing literature, which highlights a higher prevalence of bullying in outpatient departments compared to surgical wards (Johnson 2018; Sari et al. 2023). The higher rates of bullying in outpatient settings may be attributed to the stressful work environment, high patient turnover, and limited peer support, which can increase vulnerability to bullying (Williams 2016; Johnson 2018).

### 4.1 Limitations

This study used self‐reported data collection instruments; hence, we were able to evaluate nurses' reports and perceptions, but we did not obtain data from direct observations of the nurses while at work to ascertain the presence of bullying. The study was limited to only three hospitals in the region and, therefore, may not be generalisable to the entire country of Ghana. Additionally, the study focused solely on bullying among nurses, excluding other team members; thus, the overall extent of bullying in the workplace may be underestimated.

### 4.2 Implications of Study's Findings

Workplace bullying is a measurable issue that negatively affects nurses' mental health, job satisfaction, and performance. Therefore, nursing leaders and institutions should organise regular sensitisation programmes to raise awareness of the impact of workplace bullying. This implies that nurses working in various hospitals should be comprehensively trained on what constitutes workplace bullying. Emphasis should be placed on ways to communicate politely and effectively among themselves.

Additionally, hospital management should consider setting up disciplinary committees to address bullying, encourage nurses to report incidents, penalise offenders, and protect those who either witness or are being bullied. Consideration may also be given to enacting specific laws on nurse safety at the national level. With support from legislation, organisational and institutional policies, and education, nursing staff can provide care in environments free from bullying.

Further research may be conducted to determine the prevalence of workplace bullying among nurses working in other regions of the country, particularly in hospitals with a higher male‐to‐female ratio. The perspectives of nurses on workplace bullying may also be further explored using a qualitative approach. We believe this is critical to ensuring the delivery of quality patient care.

## 5 Conclusion

The study revealed that the majority of nurses had witnessed workplace bullying, with a high prevalence among nurses in hospitals and wards. A considerable number had also been victims of such bullying. Negative behaviours such as having one's opinions ignored were frequently observed, while the daily spreading of gossip and rumours was the most commonly reported behaviour. Female nurses were more often both perpetrators and targets, with senior staff commonly acting as perpetrators and junior staff as targets. Furthermore, a significant association was found between department and the perceived prevalence of workplace bullying, with nurses in surgical wards experiencing lower odds of bullying compared to their colleagues in the Outpatient Department. The study also highlighted that workplace bullying led to intentions to migrate for work, feelings of powerlessness, strained working relationships, and inferiority complexes among nurses. These findings underscore the urgent need for interventions to address workplace bullying in the nursing profession to safeguard nurses' well‐being and improve the overall work environment.

## Author Contributions

The Authors listed in the manuscript contributed significantly to the conception and design of the study and therefore merit authorship.

## Conflicts of Interest

The authors declare no conflicts of interest.

## Data Availability

The data used for this study is available on request from the corresponding author.
